# Balancing benefits and burdens: a systematic review on ethical and social dimensions of gene and cell therapies for hereditary blood diseases

**DOI:** 10.1186/s12910-025-01188-3

**Published:** 2025-03-14

**Authors:** L. C. van Hooff, E.-M. Merz, A. S. Kidane Gebremeskel, J. A. de Jong, G. L. Burchell, J. E. Lunshof

**Affiliations:** 1 Department Research & Lab Services, Donor Studies Group, Sanquin Blood Supply Foundation, Amsterdam, the Netherlands; 2 Department of Sociology, Vrije Universiteit Amsterdam, Amsterdam, the Netherlands; 3 Department of Hematology, Erasmus Medical Center, Rotterdam, the Netherlands; 4 Medical Library, Vrije Universiteit Amsterdam, Amsterdam, the Netherlands; 5 Department of Genetics, Wyss Institute for Biologically Inspired Engineering at Harvard & Harvard Medical School, Boston, MA USA

**Keywords:** Hereditary blood disease, Sickle cell disease, Diamond-Blackfan anemia syndrome, Anemia, Cell and gene therapies, Availability, Acceptability, Accessibility, Affordability, Responsible research and innovation

## Abstract

**Background:**

Sickle cell disease (SCD) and Diamond-Blackfan anemia syndrome (DBAS) are two hereditary blood diseases that present significant challenges to patients, their caregivers, and the healthcare system. Both conditions cause severe health complications and have limited treatment options, leaving many individuals without access to curative therapies like hematopoietic stem cell transplantation. Recent advancements in gene and cell therapies offer the potential for a new curative option, marking a pivotal shift in the management of these debilitating diseases. However, the implementation of these therapies necessitates a nuanced understanding of the ethical and social implications.

**Methods:**

In this mixed methods systematic review, we explore the responsible development and implementation of gene and cell therapies for SCD and DBAS and aim to sketch a path toward ethically and socially sound implementation. Drawing upon principles of Responsible Research & Innovation and the 4A framework of availability, accessibility, acceptability, and affordability, we thematically analyze existing research to illuminate the ethical and social dimensions of these therapies. Following established PRISMA and JBI Manual guidelines, a search across multiple databases yielded 51 peer-reviewed studies with publication dates ranging from 1991 to 2023.

**Results:**

Our thematic analysis shows that the theme of acceptability is heavily shaped by interactions between patients, caregivers, healthcare professionals and researchers, influencing treatment decisions and shaping the development of curative gene and cell therapies. Despite the generally positive perspective on these therapies, factors like the limited treatment options, financial constraints, healthcare professional attitudes, and (historical) mistrust can impede stakeholder decision-making. While acceptability focuses on individual decisions, the themes of availability, accessibility, and affordability are interconnected and primarily driven by healthcare systems, where high research and development costs, commercialization and a lack of transparency challenge equitable access to these therapies. This diminishes the acceptability for patients, revealing a complex interdependence of the themes.

**Conclusions:**

The findings suggest the need for improved communication strategies in clinical practice to facilitate informed decision-making for patients and caregivers. Policy development should focus on addressing pricing disparities and promoting international collaboration to ensure equitable access to therapies.

This review has been pre-registered in PROSPERO under registration number CRD42023474305.

## Introduction

Sickle cell disease (SCD) and Diamond-Blackfan anemia syndrome (DBAS) are two rare^1^ inherited blood diseases that have posed distinct yet overlapping challenges to patients, their caregivers, and the healthcare system for decades. SCD is characterized by the production of abnormal hemoglobin, leading to distorted red blood cells that can block blood flow and cause severe pain and organ damage [3, 4]. Due to the evolutionary advantage of the sickle cell trait in providing protection against malaria, SCD primarily affects people of color in malaria-endemic regions such as sub-Saharan Africa, but has also spread to non-endemic regions through forced and voluntary population migration [3]. DBAS on the other hand, is a bone marrow failure disorder, resulting in insufficient red blood cell production due to impaired erythropoiesis, congenital anomalies, and an increased susceptibility to cancer [5–7]. Both conditions can cause severe health complications, including chronic anemia, developmental delays, organ failure, and a reduced life expectancy [3, 5].

Therapeutic options for SCD include blood transfusions, pain management, medications to boost fetal hemoglobin production, and hematopoietic stem cell transplantation (HSCT) [3, 4]. Similarly, DBAS treatments rely on blood transfusions, corticosteroids, and HSCT [5–7]. These interventions are far from ideal as blood transfusions can cause deadly iron overload [3, 6, 7], and both blood transfusions and HSCTs are limited by donor scarcity, especially for ethnically diverse patients [4, 8]. Corticosteroids can cause growth defects and hormonal imbalances [5], while HSCT faces challenges with eligibility, graft rejection, future infertility, and immunosuppression [3–6]. Furthermore, the logistical and financial barriers to these treatments exacerbate healthcare inequities for patients on a global scale [9]. The shared reliance of SCD and DBAS on complex, insufficient and inaccessible treatments underscores broader issues in rare inherited blood disorders.

In recent years, the field of medicine has witnessed promising advancements in gene and cell therapies, offering the potential for a cure to these debilitating diseases. Therapies such as gene edited stem cell transplants hold the promise of correcting the genetic defects underlying SCD and DBAS, offering a curative approach rather than merely alleviating symptoms. An example is the gene therapy named Casgevy® (exagamglogene autotemcel) that uses CRISPR-cas9 technology to alter the genes causing SCD in bone marrow material of patients [10]. This highly innovative therapy has recently been approved by regulators in the United Kingdom, the United States of America and Europe, and opens the door for similar treatments in the near future [11–13].

While gene and cell therapies present a promising step forward, because of their novelty long-term follow-up data remain limited and techniques require further refinement [14, 15]. Nonetheless, they are transitioning from conceptual possibilities to curative options for hereditary blood diseases [16]. This shift underscores the pressing need for ethically and socially responsible implementation, ensuring these therapies are not only scientifically robust, but also acceptable, accessible, and affordable [11, 17, 18]. Particularly for rare disease populations that are disproportionally reliant on emerging therapies as they have few treatment options, and where marginalization hinders progress [16]. This is notably evident in SCD where affected individuals have long been in the margins of adequate healthcare due to scientific and medical racism, and structural socioeconomic disadvantages [1, 9, 19–21]. Moreover, both SCD and DBAS suffer from a historical lack of awareness and research due to their rarity, despite their identification over a century ago [1, 5, 6, 21–24].

Given these challenges, this mixed methods systematic review aims to explore the existing body of knowledge encompassing the responsible development and implementation of gene and cell therapies for SCD and DBAS. Addressing these complexities requires a nuanced approach to implementation, balancing scientific innovation with the broader societal need for equity. By synthesizing the existing research, we seek to shed light on the ethical and social implications of gene and cell therapies for SCD and DBAS, while providing insights that sketch a path toward ethically and socially sound implementation across broader contexts.

## Background

We focus on both gene and cell therapies, as the limited implementation of gene therapies thus far can benefit from the insights gained from existing knowledge about cell therapies. Currently, HSCT is the only curative cell therapy to patients with SCD and DBAS, but its application remains limited due to the significant risks mentioned before [3, 25]. The survival rate of HSCT for both SCD and DBAS is around 90% in children, but is significantly lower in teenagers and adult patients [3, 25]. While gene therapies eliminate the risk of graft-versus-host disease and resolve the problem of the lack of donors, they introduce other risks such as off-target effects, toxicity, and a potential increase in cancer risk, for which long-term follow-up data is still lacking [14, 15, 26]. Both gene and cell therapies share the uncertainties of immunogenicity, an unsuccessful outcome and the long-term effects of chemotherapy. Since the field of applied gene therapy is still in its infancy, combining relevant findings from cell therapies like HSCT with those from gene therapy provides valuable insights into the decisions made by patients, caregivers, and healthcare professionals. Recognizing these parallels sheds light on the factors we must address when navigating the ethically and socially responsible implementation of curative gene therapies in the near future. We use the term ‘cell therapy’ to refer specifically to studies addressing HSCT (or the older approach of bone marrow transplantation), and ‘gene therapy’ for studies discussing any form of therapeutic genetic modification.

In our research, we utilize the European principles for Responsible Research & Innovation (RRI) [27] as a basis for exploring ethically and socially responsible implementation of curative gene therapies. These principles state that in research and the implementation of cutting-edge technology, stakeholders should be involved to ensure that the outcomes align with general norms and values [28]. In this review, we operationalize this principle by using the 4A framework.

The 4A framework was developed in the course of a collaboration between co-author JEL and organizations of patients affected by genetic disorders in the Netherlands, at the end of the 1980s [29]. At that time, the focus was on clinical genetics and the 4A’s referred to the availability, accessibility, acceptability, and affordability of genetic services providing counseling and diagnostics, as an ethical demand by patients and their caregivers to enable informed decision-making in life and family planning. At that time, the 4A’s could also be applied as criteria to the (still) limited range of therapeutic options for genetic disorders, as only few therapies were available, e.g. special diets or drugs for metabolic disorders.

The 4A framework is broadly applicable to biomedical research and clinical practice and can be particularly useful in the analysis of a multi-stakeholder context. They are interdependent and each criterium is relevant for all stakeholders from a different vantage point. Accessibility presupposes availability: research by biomedical researchers must have succeeded in making a technology or drug available and only then accessibility to healthcare professionals and patients becomes a concrete topic. However, acceptability by the target group – in the case of therapies these are the patients – already plays an important role in the research design: if a novel technology is not acceptable for the target group, the research design may need to be changed, or in rare cases abandoned. Acceptability is influenced by many factors: anticipated accessibility is one, and the anticipated affordability is another. Besides patients, stakeholders in affordability are other payors: insurers and health care systems. The latter are shaped by health care governance and ultimately subject to political decision-making.

The 4A framework provides a tool for the interpretation of the interdependence of elements in biomedical research and healthcare innovation. All elements can be assessed under an ethical, social, and legal lens. In the case of gene therapy this interdependence is apparent in the literature, from the early days in the 1980s till today [30, 31]. We therefore applied the criteria from the 4A framework to guide the systematic review of the literature.

## Methods

Throughout this systematic review, we adhere to the Preferred Reporting Items for Systematic Reviews and Meta-Analyses (PRISMA) guidelines [32]. The research question, search strategy, inclusion and exclusion criteria, study selection process, critical appraisal methods, and data synthesis techniques were all predefined in a protocol registered in the preregistration platform PROSPERO [33] under registration number CRD42023474305 and conducted accordingly. The protocol including the search strategy, all included studies, the data extraction and analysis tables, and the codebook for the thematic analysis used in this systematic review can be found on the research platform Open Science Framework [34].

## Search strategy

The systematic literature search was conducted from June 6th to July 3rd, 2023, using the databases PubMed, Scopus, Clarivate Analytics/Web of Science Core Collection, and Cumulative Index to Nursing and Allied Health Literature (CINAHL). The search strategy involved combining indexed terms and free text terms for (synonyms of) ‘responsible’ (including ‘ethics’, ‘accessibility’, ‘affordability’), ‘novel techniques’ (including ‘gene therapy’ and ‘cell therapy’), ‘sickle cell disease’, and ‘Diamond-Blackfan anemia syndrome’ (for the complete search strategy, see our search log [34]). The search was restricted to peer-reviewed studies, but was not restricted by publication type, publication date or language. The search strategy was developed and conducted in consultation with medical information specialist and co-author GLB at the Vrije Universiteit Amsterdam.

**Fig. 1:**
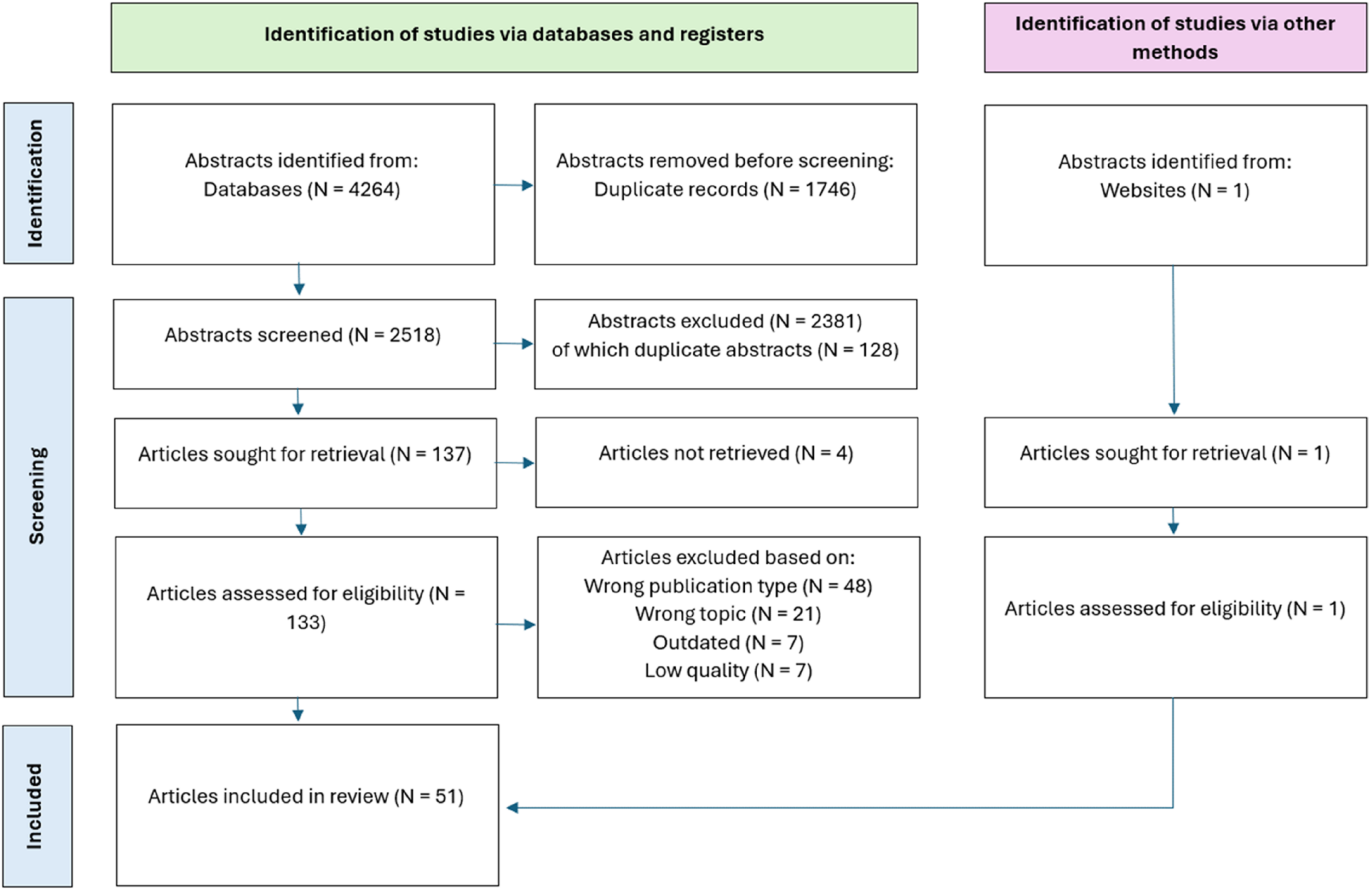
PRISMA flow diagram of paper selection process used in the present study, based on [35]

From the original search string, a total of 4,264 studies were identified. After automatic deduplication, 2,518 studies remained for title and abstract screening. During the screening process, 128 duplicates were manually flagged and excluded, and 2,253 studies were excluded based on title and abstract screening. This means that 2,381 studies in total were excluded, leaving 137 studies for full-text screening. We were unable to retrieve the full-texts of four of those studies. Based on a reference found in one of the screened full-text studies, one highly relevant study was added manually, making the total of full-text screened studies 134. After full-text screening, 51 studies were assessed as eligible, and included in the analysis. Detailed information about the study selection process is shown in the PRISMA flow diagram (Fig. 1).

### Inclusion and exclusion criteria

Studies using quantitative, qualitative or mixed methods were included if they met the following criteria. They needed to be peer-reviewed and directly address curative gene and/or cell therapies for SCD and DBAS. At the start of our screening process, we decided study designs had to encompass at least one of the 4As, i.e., availability, acceptability, accessibility, and/or affordability. Due to the large number of studies that emerged from the search and the approval of the first gene therapy for SCD during the title and abstract screening of the review [11, 13], our team decided that the question of availability became less important. Hence, studies addressing availability were only included if they also elaborated on at least one of the other A’s.

The studies should have involved one or multiple relevant stakeholders, such as patients, families of patients, healthcare professionals, researchers, and/or policymakers, within the context of SCD and DBAS. They should also either demonstrate a causal relationship between therapy and effects or explicitly discuss associations and potential causality. In studies where causality is not the focus, the directionality of associations should be clearly addressed. Lastly, there were no language, study design, publication type or publication date restrictions.

**Fig. 2:**
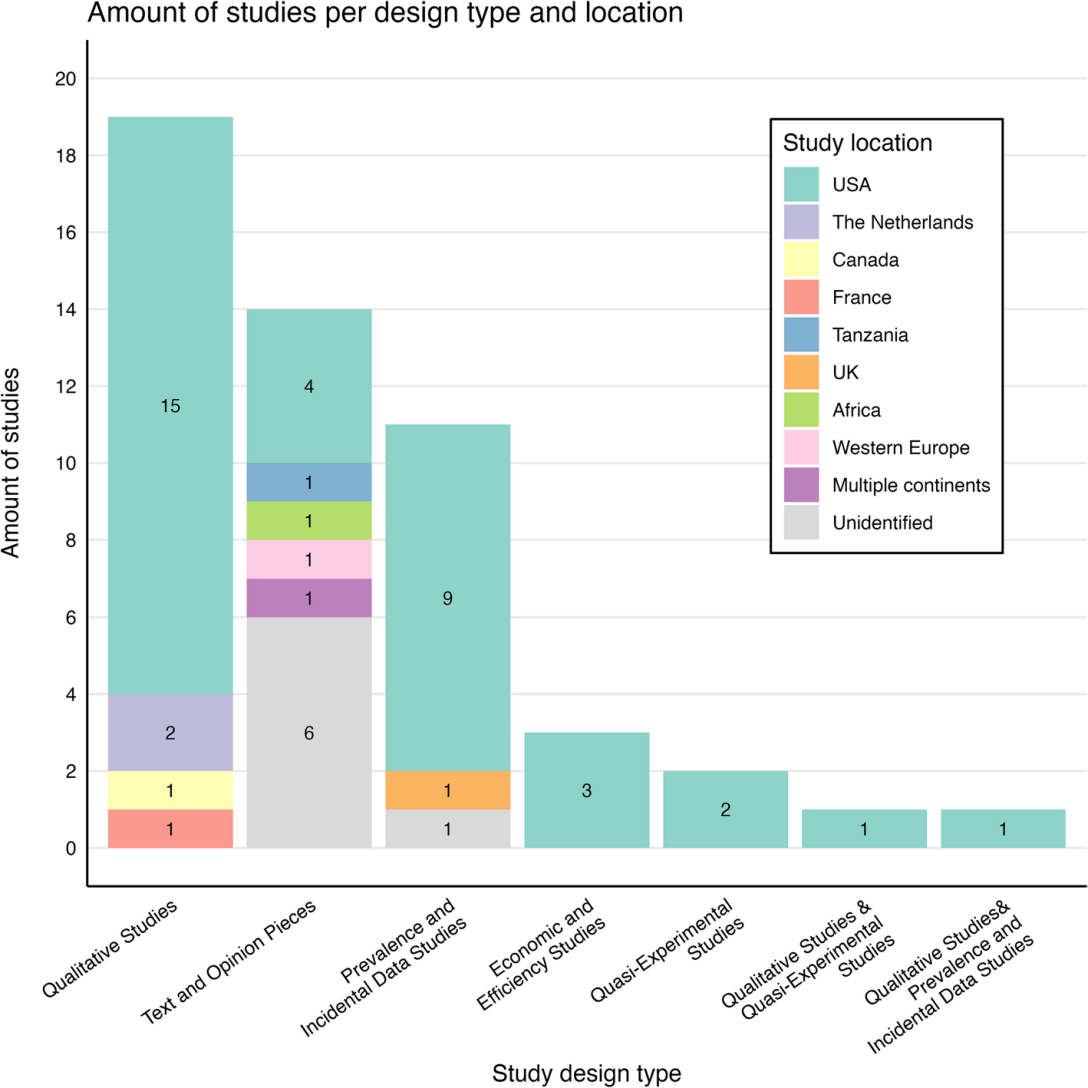
Graph showing the amount of studies per design type and study location

Ultimately, the sample included a diverse range of study designs, including qualitative research (e.g., interviews, experiences, observations), prevalence and incidental research data (e.g., surveys, large datasets), quasi-experimental studies (e.g., clinical trials), economic and efficiency research (e.g., cost–benefit studies), and text and opinion pieces (e.g., editorials, commentaries, white papers). All the studies included for full text screening were in English. Studies that were categorized as a review or abstract, or those whose quality was assessed as below moderate, were excluded (see [34]). All the studies included for analysis were published between 1991 and 2023. Information about the amount of studies included for analysis per design type and location are shown in the graph (Fig. 2).

### Study selection

Each study from the original search string was assessed for eligibility, based on title and abstract screening by author LCH and co-author EMM using Rayyan, a web app for exploring and filtering eligible studies [36]. To make the title and abstract screening process more efficient and less time consuming, the screening was not conducted in a blind manner. This means that reviewers were able to see each other’s decisions, and that a probability exists of a bias in their assessment. However, the full-text screening was done in a blind manner. Reasons for exclusion of a study were documented by all reviewers in both title and abstract screening and full-text screening. Discrepancies were resolved through discussion or third-party adjudication. As previously stated, a total of 51 studies were included in the final analysis after full-text screening.

### Quality control and data extraction

An Excel-based overview containing all included full-text studies (n = 134) was developed in which both the quality control and the data extraction for full-text screening could be reported by the review team. Firstly, the quality of full-text studies was assessed by each reviewer based on predefined criteria, using the critical appraisal tools and guideline of the JBI^2^ Manual for Evidence Synthesis [37, 38]. Data extraction was only conducted from studies meeting moderate or high-quality standards and based on predefined elements reported in the protocol. The full-text evaluation and data extraction of the studies were conducted by LCH, EMM, co-author ASKG and co-author JAJ and each full-text study was evaluated and subsequently extracted by at least two of them. Discrepancies were resolved through discussion after completion of the evaluation and extraction, and in cases of persistent disagreement, a third-party in the review team was consulted to adjudicate.

### Data synthesis and analysis

A narrative synthesis approach was employed to summarize findings about any of the 4A’s of the included quantitative, qualitative and mixed methods studies. For the narrative synthesis, the reviewers were encouraged to summarize relevant parts of the discussion and conclusion of the included studies. Results across included studies were synthesized in a table. These findings were further analyzed by LCH using thematic analysis [39] to uncover recurring themes across the different synthesis. The program ATLAS.ti was used to conduct the thematic analysis. The authors, titles and narrative syntheses of the included studies were grouped based on the relation of the study to the 4A’s (as decided by the reviewers) in separate Excel-documents each titled with ‘Availability’, ‘Acceptability’, ‘Accessibility’, or ‘Affordability’, and uploaded to ATLAS.ti. Those documents were first thoroughly read and then coded based on topics that were found in at least two narrative syntheses (see 34) After a new code emerged, all documents were skimmed again, to apply this new code elsewhere if necessary. After coding was finished, the codes were grouped in bigger overarching themes. At two points during the analysis, the codes, code groups, and final themes were discussed with EMM and JEL. Their feedback was incorporated into the analysis.

## Results

Through the thematic analysis two general main themes emerged: the first being ‘Stakeholder interaction: acceptability of patients, caregivers and healthcare professionals of curative gene and cell therapies’. The second theme, ‘International availability, accessibility and affordability of gene and cell therapies’, mostly covers the frame of the other three A’s. Codes and subcodes identified through the thematic analysis and organized under the two main themes are accentuated in italics (for a full overview of the themes and how they emerged from the codes and subcodes, see [34]).

During the screening processes, it already became apparent that very little has been written on DBAS, let alone from a social and ethical perspective. After title and abstract and full-text screening, only two expert opinion pieces on DBAS were included. As a result of this, the findings will almost exclusively be based on SCD studies. More research is needed to see if the results are the same for DBAS. Some of the topics that occur in the two included DBAS studies are in agreement with the findings in the SCD studies. While discussing the results, we will indicate when this is the case.

### Stakeholder interaction: acceptability of curative gene and cell therapies by patients, caregivers and healthcare professionals

During the analysis, one of the first things that stood out to us was the central role of *stakeholder interaction* on the *decision-making* process surrounding curative gene and cell therapies. While summarizing the results of mostly the qualitative and survey studies in which decision-making was a topic, the inseparable connection between the *patient* and their *caregivers* (parents, guardians and involved extended family) in relation to decision-making became visible. This was not surprising, since 38 (75%) of the included studies covered HSCT. Outcomes of HSCT are most beneficial in young SCD and DBAS patients [25, 40, 41], and in that case caregivers have to give consent for the underage patients and have the final say in treatment decision-making. However, also in studies focusing on adolescent and adult SCD patients, the involvement of loved ones in treatment decision-making was found.

For instance, if caregivers, extended family, or friends were not supportive of a patient undergoing curative therapy, SCD patients were unlikely to choose that option [41, 42]. Yet more often, it worked the other way around. One of the studies in which the involvement in decision-making of caregivers became apparent and simultaneously pointed out the necessity of taking this into account, showed how SCD patients sometimes felt pressured by their caregivers to undergo HSCT [43]. In twelve studies (24%), the strong desire to escape the complications of SCD, the side effects of the limited treatment options, and the *psychological and emotional burden* associated with SCD, were big motivators to choose a curative therapy for patients [41, 44–53], and for their caregivers [41, 42, 44, 47, 48, 50, 52, 54].

*Anxiety*, *powerlessness* and *uncertainty* were themes that recurred in the analysis when SCD patients and their caregivers talked about living with SCD [41, 43, 54, 55] and besides practicing *spirituality and religion* [40–42], looking for any possible (curative) treatment option was also mentioned as a *coping strategy* for both [41, 54]. It is important to consider the few treatment options that these patients and their caregivers have, and how ‘the hope of a cure’ might influence their decision to join clinical trials or undergo risky therapies [55].

This is not to say that the hope and optimism these stakeholders feel towards curative therapies are unjustified. Indeed, seven studies (14%) showed that DBAS patients and SCD patients that underwent curative therapy had significant *improvements in health* [25, 56–58] and *quality of life* [41, 43, 56, 59]. A theme that kept recurring was how post-HSCT SCD patients and their caregivers felt they entered a *‘new’ or ‘second chance’ on life* [40, 41, 43]. Two studies reported that even post-HSCT patients who had serious complications from the procedure, did not report decisional regret [41, 42].

Nevertheless, all SCD and DBAS stakeholders, from patients to their caregivers, to healthcare professionals, to researchers, considered the gene and cell therapies *risky* [23, 25, 41, 44, 47, 49–55, 60–62]. For some patients and caregivers the promise and benefit of a cure outweighs all the risk of a treatment, however high that risk might be [41, 42, 46, 48–50, 53, 63, 64]. However, many other SCD patients and caregivers who had a *positive perspective* on the outcome of a curative gene or cell-therapy, still showed well-considered, contextualized and nuanced risk–benefit decision-making [42, 43, 45, 48].

An example of this was found in one study in which a small sample of SCD patients could choose between multiple hypothetical curative gene and cell therapies. The patients considered all of the therapies beneficial because of the prospect of being cured and chose the gene correction therapy most often as therapy of preference [45]. However, several of them still reported feeling unlikely to apply for any of the therapies if they were available to them now, because of the high risk involved. Two other studies showed that despite SCD patients and caregivers supporting the medical use of curative gene and cell therapies more than the general population (and just as much as healthcare professionals and genetic technicians), they simultaneously felt it sometimes conflicted with their personal and moral *values* [48], which eventually could create serious decisional conflicts that in some cases even delayed treatment [51]. One study found that parents may not fully align with their children’s perspectives regarding the patient’s quality of life with SCD [47], further contributing to the complexity of caregivers’ risk–benefit assessment of therapy choices for their children [51].

Interestingly, the included studies reported contradictory findings on the influence of *disease severity* and the influence of *infertility* on the risk–benefit decision-making of treatments by stakeholders. Where four studies (9%) stated they found no relation between disease severity and a patient’s or caregiver’s decision to undergo a curative gene or cell therapy [46, 55, 64, 65], seven others (14%) did find such a relation [41, 44, 47, 50, 51, 54, 62]. Disease severity did seem to influence decision-making among healthcare professionals [54, 61, 66]. Statements on infertility were similarly inconclusive, with four studies mentioning how patients and caregivers considered the possibility of a cure worth the risk of infertility [41, 47, 49, 60], while three others found the risk of infertility unacceptable [46, 62, 67]. Whether counselling from healthcare providers on fertility preservation options changed the acceptability was debated and remained unclear, but nevertheless it was being recommended for both SCD and DBAS [25, 46, 60, 62, 67].

It is not just SCD patients and caregivers that sometimes consider the curative therapies too risky, despite the positive outcomes. Six studies (12%) showed how access to curative gene and cell therapies is gatekept by healthcare professionals. The attitudes of healthcare professionals towards the risk of curative therapies and their frame of reference and assessment of the *cultural, social and economic situation* of SCD patients and their caregivers heavily influenced their practice of discussing these therapies in a treatment plan [46, 53, 61, 66, 68, 69]. Healthcare professionals were identified by SCD patients and caregivers as their most important source of information for (experimental) treatment options [52, 70, 71]. As a result, the attitudes and perspectives of healthcare professionals influence the (access to) information SCD patients and caregivers receive and the decisions they make [68]. This could potentially lead to health inequity. The importance of multidisciplinary healthcare professional teams and standardization of a discussion on HSCT in treatment plans with patients and their caregivers are highlighted as important steps in promoting shared decision-making and removing this potential barrier to curative gene and cell therapies [69, 70].

Something else that stood out in the relation between SCD patients and their caregivers on one side and the healthcare professionals and researchers on the other side was the theme of *trust and mistrust*. Four studies emphasized the importance of a trust-based relationship between healthcare professionals and SCD patients and caregivers in making decisions on (experimental) curative therapy, mentioning that high levels of openness and dialogue lie at its basis [43, 66, 70, 71]. When considering undergoing a curative therapy or *joining clinical trials* for curative therapies, trust or mistrust in healthcare professionals and researchers create either a facilitator or a barrier for SCD patients and caregivers [42, 52, 62, 66, 70–72].

Seven studies (14%) specifically mentioned the SCD patients’ negative *history* with the medical (research) system and the people working in it as a source of mistrust^3^ [66, 70–72, 74–76]. For example, three studies described a lack of trust in researchers [42, 70, 71]. In two of those studies, SCD patients and caregivers were unwilling to join clinical trials, because historical events and prior family experiences led them to fear that researchers would be untruthful about or would conceal useful information from (minority) patients [70, 71]. One study expressed patients and caregivers fearing that their community would be excluded from the long-term benefits of the research [71]. They assumed the industry would ultimately go for profit instead of equal access once the therapies would become reality. This mistrust forms a barrier to join clinical trials, meaning that stakeholder’s perspective on *accessibility* and *affordability* could have direct implications for the *availability* of the therapies.

Despite the possible barrier of mistrust, six studies (12%) did find a willingness in their sample of SCD patients and caregivers to join clinical trials for curative gene and cell therapy, but mentioned that patients and caregivers simply did not know where to find *information* on those, or whom they could inform about their interest to participate [53, 54, 62, 70–72]. The same seems true for undergoing HSCT. Seven studies (14%) found that patients and caregivers often were aware of HSCT as a treatment, but did not know how it worked, if the option was available to them and how to apply for it [41, 46, 51, 53, 54, 65, 67], potentially creating another barrier. This lack of knowledge on HSCT was also related to healthcare professionals not discussing the options with SCD patients and caregivers mentioned earlier. In the case of DBAS, one expert opinion piece written by a caregiver of a patient stated how the absence of information, caused by the general lack of knowledge about DBAS, can immobilize families and lead to sleepless nights [23].

All these studies show how important it is for patients and their caregivers to be informed as much as possible about their disease and their treatment options. Still, one study mentioned that the health literacy and knowledge of SCD patients and caregivers in relation to SCD and curative gene and cell therapies might be underestimated, since they have been dealing with the disease and medical field since the diagnosis [72]. Underestimating the knowledge of patients and caregivers could also lead to mistrust between stakeholders and should be avoided [72].

The solutions offered for the problems of mistrust and lack of knowledge of SCD patients and caregivers, were: developing a good relationship with the healthcare professionals and researchers, detailed and longitudinal information sharing, and a long-term process of gathering informed consent about curative gene and cell therapy and clinical trials [52, 53, 62, 70–72]. SCD patients and their caregivers need to feel listened to and taken serious as conversation partners by healthcare professionals and researchers, not to be pressured into make a certain choice, nor feel pressured to make that choice quickly [43, 52, 53, 62, 70–72]. Information should be provided in a neutral but elaborate way over multiple occasions, by different experts and preferably in person. Folders, images and videos, a small-sized conference, discussion with patients that previously underwent the therapy, and an online decision-making aid were also used to help convey information, and were all positively welcomed by SCD patients and caregivers [52, 67, 77]. Moreover, to ensure both SCD patients and their caregivers are assisted according to their specific social and psychological needs throughout the patients’ clinical pathways, and especially during and after the challenging process of undergoing a curative gene or cell therapy, five studies (10%) explicitly mentioned the necessity for psychological support and social work in the healthcare team [40, 46, 52, 56, 59, 78].

The findings in this section highlight how the acceptability of a curative therapy for each patient and caregiver should be seen in their own context, and that the different stakeholders do not operate in a social vacuum. The influence of the exchange of perspectives and values between patients, their caregivers, the healthcare professionals and even researchers, should be taken into consideration when looking through the frame of acceptability. This has implications for the implementation of gene and cell therapies, which we will attend in the discussion section. As we have already seen through the findings of Omondi et al. [70], stakeholder perspectives also play a role in the advancement and equitable distribution of gene and cell therapies. Following the exploration of acceptability and stakeholder perspectives on these therapies, we therefore now shift our focus to availability, accessibility, and affordability.

### International availability, accessibility and affordability of gene and cell therapies

Throughout the analysis, it became apparent that the questions of availability, accessibility and affordability are interconnected and heavily interdependent. We will address this in the next section, starting with the recurrent theme of *costliness of gene and cell therapies*. This was mentioned in two studies on an individual level by stakeholders, with some USA-based SCD patients and caregivers explaining they encountered difficulties with paying for HSCT [41], but also by USA-based healthcare professionals that did not discuss HSCT with some of their SCD patients and caregivers due to their *financial situation* [61]. Yet eight studies (16%) mentioned expensiveness on national and global levels, stating that the (assessed) prices of curative gene and cell therapies for SCD and other rare diseases (like DBAS) would have a considerable impact on national healthcare budgets and therefore it would probably come down to patients and caregivers paying a large amount of the therapy themselves [73, 76, 79–82]. One study mentioned different ways in which reimbursement for the high-priced therapies could be tackled to make it more feasible for patients and insurance companies, specifically naming annuity payment models and outcome-based contracting [73]. However, the study also states that annuity payment would still not be beneficial from a societal perspective, and experience with outcome-based contracting is limited and might impede large-scale implementation in a US setting [73]. Moreover, the therapies would still be unaffordable and therefore inaccessible in healthcare systems of the low and middle-income countries where SCD is most prevalent [79, 83, 84].

According to five studies (10%), the accumulated reasons behind the extremely high prices set by pharmaceutical companies include the research, *development,* and manufacturing costs, the related expenses of the specific disease, the societal and economic benefits of reducing disease burden, the long-term health and quality of life improvements for patients, and the level of personalization of the therapy [73, 79–81, 85]. Additional factors mentioned to increase the price of the therapies were market dynamics, a lack of competition, and price negotiations between countries and pharmaceutical companies taking place behind closed doors [79, 82]. Due to commercialization and the absence of strong competition, the process of calculating a price for these therapies is not disclosed, leaving a lot of room for profit to the pharmaceutical companies [79, 82].

Two included opinion pieces stated that the price should reflect the tax-payer money that formed the basis of research done into curative gene and cell therapies [79, 82]. Four other studies explicated how unfair the pricing of these therapies is in relation to the *historical health disparity* of SCD patients and built cases to let this disparity be reflected in the price as there is ‘a debt to be paid’^4^ [56], see also [61, 64, 66]. Solutions that have been offered to reduce the expensiveness of curative gene and cell therapies are mostly found in more *international and interdisciplinary collaboration*, governmental intervention, and simply in more research and development of curative gene and cell therapies in different healthcare systems, to increase competition and treatment options [79, 83, 84, 86, 87].

Four studies argued that curative gene and cell therapies could be developed on a local level in low and middle-income countries with the help of international partnerships with established healthcare and research institutions via on-site involvement during the setup, eventually followed by online interactions and support [83, 84, 86, 87]. This local development could potentially lower the price in low and middle-income countries and hence bring more distributive justice. One study mentioned that in the case of curative gene and cell therapies for SCD in Africa, price negotiations with pharmaceutical companies could potentially be done through the African Union which would be ‘…negotiat[ing] on behalf of 1.3 billion people’ [83]. This would make the position to negotiate stronger than for individual countries.

The findings in this section mostly tap into the frame of affordability, but also consider its connection with the availability and accessibility of curative gene and cell therapies across diverse healthcare contexts. In summary, there is a global concern regarding the current high costs of these therapies, posing significant barriers to accessibility for various healthcare systems and stakeholders. Localized research and development efforts emerge as a potential solution to improve availability, accessibility, and affordability on a global scale, but the need for coordination at an international level becomes apparent.

## Discussion

Curative gene and cell therapies hold immense promise in addressing genetic disorders like SCD and DBAS. However, the responsible development and implementation of these therapies necessitate adherence to ethical principles, such as those outlined in the European guidelines for RRI. In this mixed methods systematic review, we operationalized the RRI principles by employing the 4A framework of availability, acceptability, accessibility, and affordability to explore the social and ethical horizons of curative gene and cell therapies for SCD and DBAS. By doing so, we aim to shed light on the considerations inherent in the advancement and implementation of these therapies.

### DBAS

It is necessary to first acknowledge the lack of studies exploring the 4A’s in the context of curative gene and cell therapies for DBAS. DBAS is a rare genetic disorder and therefore remains understudied, from a medical and a patient perspective [7, 88]. Compared to SCD, DBAS has not benefitted from the same level of societal attention, likely due to its significantly smaller patient population [5, 88] and the absence of advocacy and awareness campaigns driven by historical inequities (as is the case with SCD, see for example [89]). Furthermore, the limited population size for rare disease therapies reduces economic incentives for comprehensive research, including clinical trials and the exploration of social and ethical dimensions [7, 90, 91].

Due to the rarity of the disease, DBAS patients and their caregivers face unique healthcare needs and challenges that need to be studied [6, 88]. Without research specifically addressing the needs of patients with rare diseases like DBAS, there is a risk of overlooking important factors influencing treatment decisions, access to care, and the successful implementation of emerging therapies [7, 92]. These gaps emphasize the need for future research efforts to address both the medical challenges and the social and ethical dimensions of DBAS, especially now that gene therapy emerges as a potential therapeutic option [15, 24]. Our analysis provides various valuable insights on the 4A’s of curative gene and cell therapies for SCD, which may serve as a foundation for addressing similar challenges in other rare hereditary blood diseases like DBAS.

### Acceptability

During the thematic analysis, the frame of acceptability was broadly addressed in the included studies under the theme of stakeholder interaction. Our analysis of the included studies reveals the interplay between firstly patients and their caregivers [41, 43], but also between them and their healthcare professionals [46, 53, 61, 66, 68–70], and even researchers [62, 71, 72] in shaping both treatment decisions and research development. On first sight, the mostly positive perspectives of different stakeholders are useful for the implementation and uptake of curative gene and cell therapies [42, 46]. However, these studies only reflect perspectives from Northern America and Western Europe (see Fig. 2), highlighting a gap in the literature regarding the acceptability of these therapies in other healthcare contexts. This calls for further research into factors influencing patient and caregiver acceptability of the therapies in diverse settings.

At the same time, our analysis underscores the importance of considering the contextual factors that influence patients’ and caregivers’ decision-making, even within the Northern American and Western European perspective. These include the lack of a real choice due to limited treatment options, external and internal pressure to undergo a curative therapy if the opportunity presents itself, financial constraints, and specifically in the case of SCD (historical) mistrust between stakeholders [41–43, 66, 70]. This mistrust is deeply rooted in systemic and interpersonal racism that SCD patients and caregivers have experienced [19–21, 93], which was only explicitly addressed in one of the included studies [73]. While other studies hinted at racism by referencing terms like ‘mistrust towards healthcare professionals and researchers due to personal and historical events’ (see for instance [17, 66, 70]), they did not directly frame these dynamics as a result of racism. This lack of explicit recognition obscures the structural nature of these inequalities and hinders the development of equitable, trust-building solutions that address disparities and respond to the lived experiences of patients and caregivers.

Additionally, factors such as disease severity, concerns about infertility, and a lack of knowledge or information about their condition, existing treatment options, and research into new therapies could play a role. However, the included studies were inconclusive on these topics [41, 46, 49, 55, 60, 62, 65–67]. For healthcare professionals, the factors that influence the treatment decision-making were their own attitudes towards the curative gene and cell therapies, their evaluation of the patient’s disease severity, and their assessment of the social and economic situation of patients and their caregivers [53, 61, 66, 68, 69].

Finally, we want to emphasize the repeated calls for greater psychological and social support from healthcare teams during and after the incredibly difficult process of undergoing a curative therapy [40, 46, 52, 56, 59, 78]. This need is particularly critical, given the essential use of chemotherapy and the following period of isolation due to a compromised immune system, as well as the challenges of accustoming to a ‘new’ life [41, 56, 59]. Providing such support, could potentially also increase the trust in healthcare professionals, as the patients and caregivers could feel more listened to and looked after [94].

The findings raise hard questions like: how can healthcare providers communicate the risks and benefits of curative therapies to patients and caregivers in ways that avoid bias and foster trust, understanding, and informed decision-making? How can we ensure that patients and caregivers are provided with clear, accessible information about their condition and treatment options, while respecting their life-long personal experience with the disease and the medical system? What strategies can be implemented to ensure that patients and their caregivers provide fully informed consent for both participating in clinical trials and undergoing treatments? Finally, how can the issue of systemic racism and the lack of diverse healthcare context research be addressed to rebuild trust, improve healthcare access, mitigate health disparities, and explore the acceptability of non-Western patients and caregivers?

To ethically and responsibly implement curative gene and cell therapies, we need to put patient autonomy front and center, without losing track of patients’ embeddedness in a social network and context. Ways to address the barriers to stakeholder acceptability and accessibility are through open and longitudinal information-sharing, working on a good personal bond between stakeholders and establishing shared-decision making in multidisciplinary healthcare teams [43, 62, 69–72].

### Availability, accessibility and affordability

The frames of availability, accessibility and affordability were less prominent in the thematic analysis than the frame of acceptability. This could possibly be due to the inclusion restriction of peer-reviewed studies, which automatically brings an almost solely ‘academic’ perspective. ‘Non-academic’ policy documents could potentially cover more information on availability, accessibility and affordability from governmental and industry perspectives. It is also noteworthy that most of the included studies referred to in this section were USA-based (see Fig. 2). Both points underscore the necessity for more original research into the availability, accessibility and affordability of curative gene and cell therapies in different healthcare systems to inform evidence-based policy on a more global scale.

The frame of affordability was most prominently reflected in the analysis under the theme of expensiveness, which in turn was heavily interconnected to the frames of availability and accessibility. Discussions on the price of curative gene and cell therapies have been held from micro to macro level. The extremely high cost of these therapies have a great impact on the healthcare budgets of countries and of patients and families, leading to large differences in accessibility depending on the healthcare context. Pharmaceutical companies justify the high prices by referring to the years of costly research and development that preceded the therapies, by factoring in the long-term health and productivity outcomes of SCD patients, and by the high degree of personalized treatment that precludes mass production [73, 79–81, 85]. Yet, due to the commercialization and lack of competition in the availability of the therapies, a profit motive is also at play [79, 82].

While included studies noted several contributing factors to the high costs of these therapies, none provided a detailed or comprehensive breakdown of the pricing structures and cost calculation practices. This reflects broader challenges in the field, where proprietary and non-disclosed processes limit public understanding of cost calculation and leave significant room for profit by pharmaceutical companies [95]. Since the UK, USA and EU approval of gene therapy for SCD in November 2023, the debate on affordability and health disparity has gained even more momentum [11, 12, 95–100]. More detailed analyses of price setting and reimbursement pathways have been conducted and different pricing and payment structures based on various ethical perspectives have since been proposed [95, 98, 100]. Our analysis sheds light on the currently present and expected future disparities in access to curative gene and cell therapies across different healthcare systems if the price continues to be this high.

The distribution of healthcare resources, and the pricing of therapies raise profound ethical questions, such as: what ethical principles should guide the pricing of curative gene and cell therapies? And (how) should scarce healthcare resources be allocated to maximize public health benefits and promote social justice? These questions highlight tensions between different ethical frameworks: utilitarian healthcare policies aim to maximize overall societal benefits, while deontological approaches emphasize the moral obligation to prioritize individuals in need, even if it challenges broader utility [95, 101].

We wish to argue that researchers, healthcare professionals, policymakers, and pharmaceutical companies, bear responsibility in ensuring justice and beneficence in the development and implementation of these therapies, because their decisions directly influence aspects such as research priorities, approval processes, pricing strategies and equitable distribution. Their expertise and authority grant them the power to shape outcomes that impact accessibility and fairness of curative therapies. Ways to do this include advocating for policy changes and conditions of patenting and licensing, letting the prices reflect the public investments that have been made in the research and development process, and specifically targeting healthcare disparities [76, 79, 82, 102]. Moreover, increasing international collaboration on research efforts in the development of curative gene and cell therapies could influence both availability and affordability, which in turn will create more accessibility [79, 82, 84].

### Strengths, limitations and future perspectives

While our study provides valuable insights into the availability, acceptability, accessibility and affordability of curative gene and cell therapies for SCD and DBAS it is essential to acknowledge strengths and limitations of our research. By employing a thorough reviewing process and thematic analysis and considering a wide array of sources, including expert opinion pieces and empirical studies, we were able to provide a comprehensive overview of stakeholder perspectives. This approach facilitated the identification of novel insights into decision-making dynamics, acceptability, availability, accessibility and affordability to curative therapies. The practical recommendations derived from our review potentially offer steps for policymakers and practitioners to enhance the acceptability, availability, accessibility, and affordability of these therapies in the field.

However, while our focus on stakeholder perspectives provided rich insights into the specific context of curative therapies for SCD and DBAS, it may limit the generalizability of our findings to other genetic conditions. Future research endeavors could aim to explore a broader range of conditions and settings to enhance the applicability of the insights generated. Additionally, the inherent subjectivity and potential bias introduced by narrative synthesis and thematic analysis should be acknowledged, despite efforts to mitigate these through rigorous analysis, review team discussions and reflexivity. Moreover, the reliance on peer-reviewed studies may have constrained the depth and scope of our analysis. As discussed earlier, the inclusion of non-academic policy documents and white papers, might provide further insights.

Furthermore, several other avenues for future research warrant exploration. The necessary cut-off date for the collection of studies, inevitably led to the exclusion of publications on groundbreaking developments after that date. Future investigations could delve into the experiences and long-term outcomes of SCD and DBAS patients and caregivers undergoing these therapies and evaluate the effectiveness of interventions aimed at improving access and equity. As stated earlier, research into different healthcare settings across the world could also bring new insights for availability, acceptability, accessibility and affordability, and could provide ways to improve international collaboration.

In conclusion, our systematic review sheds light on the multifaceted social and ethical considerations encompassing curative gene and cell therapies for SCD and DBAS. By understanding the perspectives of stakeholders and addressing the ethical and practical challenges they present, we can strive towards more responsible, equitable and patient-centered approaches to healthcare delivery in the era of precision medicine.

## Data Availability

The datasets generated and analyzed during the current study are available in the OSF repository with DOI 10.17605/OSF.IO/SBRNG, which can be found on https://osf.io/sbrng/ and are also available from the corresponding author upon reasonable request.

## Abbreviations

SCD: Sickle cell disease
DBAS: Diamond-Blackfan anemia syndrome
RRI: Responsible Research & Innovation
HSCT: Hematopoietic stem cell transplantation
PRISMA: Preferred Reporting Items for Systematic Reviews and Meta-Analyses
CINAHL: Cumulative Index to Nursing and Allied Health Literature
4A: Availability, acceptability, accessibility and affordability
USA: United States of America
EU: European Union
UK: United Kingdom

## Footnotes

1 It is important to note that SCD is considered a rare disease in Western high-income countries, leading to a historical lack of awareness, attention and research [1], but that SCD is not rare at all on a global scale [2].

2 Formerly known as the Joanna Briggs Institute.

3 Interestingly, only one of the included studies explicitly mentioned interpersonal and systemic racism towards SCD patients [73], and this study was not focusing on patient and caregiver perspectives, but on national healthcare budgets for gene therapy. Instead other studies leaned more towards terminology like ‘mistrust due to personal and historical events’. Some of them did hint towards racism (see for instance [17, 66, 70].

4 It is important to repeat here that the inaccessibility and unaffordability of curative therapies to the SCD community was mentioned as a fear and possible barrier by SCD patients and caregivers to join clinical trials [70]
